# A critical assessment of using ChatGPT for extracting structured data from clinical notes

**DOI:** 10.1038/s41746-024-01079-8

**Published:** 2024-05-01

**Authors:** Jingwei Huang, Donghan M. Yang, Ruichen Rong, Kuroush Nezafati, Colin Treager, Zhikai Chi, Shidan Wang, Xian Cheng, Yujia Guo, Laura J. Klesse, Guanghua Xiao, Eric D. Peterson, Xiaowei Zhan, Yang Xie

**Affiliations:** 1 Quantitative Biomedical Research Center, Peter O’Donnell School of Public Health, University of Texas Southwestern Medical Center, 5323 Harry Hines Blvd, Dallas, TX USA 75390 USA; 2 Department of Pathology, University of Texas Southwestern Medical Center, 5323 Harry Hines Blvd, Dallas, TX USA 75390 USA; 3 Department of Pediatrics, University of Texas Southwestern Medical Center, 5323 Harry Hines Blvd, Dallas, TX USA 75390 USA; 4 Department of Internal Medicine, University of Texas Southwestern Medical Center, 5323 Harry Hines Blvd, Dallas, TX USA 75390 USA

**Keywords:** Pathology, Non-small-cell lung cancer

## Abstract

Existing natural language processing (NLP) methods to convert free-text clinical notes into structured data often require problem-specific annotations and model training. This study aims to evaluate ChatGPT’s capacity to extract information from free-text medical notes efficiently and comprehensively. We developed a large language model (LLM)-based workflow, utilizing systems engineering methodology and spiral “prompt engineering” process, leveraging OpenAI’s API for batch querying ChatGPT. We evaluated the effectiveness of this method using a dataset of more than 1000 lung cancer pathology reports and a dataset of 191 pediatric osteosarcoma pathology reports, comparing the ChatGPT-3.5 (gpt-3.5-turbo-16k) outputs with expert-curated structured data. ChatGPT-3.5 demonstrated the ability to extract pathological classifications with an overall accuracy of 89%, in lung cancer dataset, outperforming the performance of two traditional NLP methods. The performance is influenced by the design of the instructive prompt. Our case analysis shows that most misclassifications were due to the lack of highly specialized pathology terminology, and erroneous interpretation of TNM staging rules. Reproducibility shows the relatively stable performance of ChatGPT-3.5 over time. In pediatric osteosarcoma dataset, ChatGPT-3.5 accurately classified both grades and margin status with accuracy of 98.6% and 100% respectively. Our study shows the feasibility of using ChatGPT to process large volumes of clinical notes for structured information extraction without requiring extensive task-specific human annotation and model training. The results underscore the potential role of LLMs in transforming unstructured healthcare data into structured formats, thereby supporting research and aiding clinical decision-making.

## Introduction

Large Language Models (LLMs)^1–6^, such as Generative Pre-trained Transformer (GPT) models represented by ChatGPT, are being utilized for diverse applications across various sectors. In the healthcare industry, early applications of LLMs are being used to facilitate patient-clinician communication^7,8^. To date, few studies have examined the potential of LLMs in reading and interpreting clinical notes, turning unstructured texts into structured, analyzable data.

Traditionally, the automated extraction of structured data elements from medical notes has relied on medical natural language processing (NLP) using rule-based or machine-learning approaches or a combination of both^9,10^. Machine learning methods^11–14^, particularly deep learning, typically employ neural networks and the first generation of transformer-based large language models (e.g., BERT). Medical domain knowledge needs to be integrated into model designs to enhance performance. However, a significant obstacle to developing these traditional medical NLP algorithms is the limited existence of human-annotated datasets and the costs associated with new human annotation^15^. Despite meticulous ground-truth labeling, the relatively small corpus sizes often result in models with poor generalizability or make evaluations of generalizability impossible. For decades, conventional artificial intelligence (AI) systems (symbolic and neural networks) have suffered from a lack of general knowledge and commonsense reasoning. LLMs, like GPT, offer a promising alternative, potentially using commonsense reasoning and broad general knowledge to facilitate language processing.

ChatGPT is the application interface of the GPT model family. This study explores an approach to using ChatGPT to extract structured data elements from unstructured clinical notes. In this study, we selected lung cancer pathology reports as the corpus for extracting detailed diagnosis information for lung cancer. To accomplish this, we developed and improved a prompt engineering process. We then evaluated the effectiveness of this method by comparing the ChatGPT output with expert-curated structured data and used case studies to provide insights into how ChatGPT read and interpreted notes and why it made mistakes in some cases.

## Results

### Data and endpoints

The primary objective of this study was to develop an algorithm and assess the capabilities of ChatGPT in processing and interpreting a large volume of free-text clinical notes. To evaluate this, we utilized unstructured lung cancer pathology notes, which provide diagnostic information essential for developing treatment plans and play vital roles in clinical and translational research. We accessed a total of 1026 lung cancer pathology reports from two web portals: the Cancer Digital Slide Archive (CDSA data) (https://cancer.digitalslidearchive.org/) and The Cancer Genome Atlas (TCGA data) (https://cBioPortal.org). These platforms serve as public data repositories for de-identified patient information, facilitating cancer research. The CDSA dataset was utilized as the “training” data for prompt development, while the TCGA dataset, after removing the overlapping cases with CDSA, served as the test data for evaluating the ChatGPT model performance.

**Fig. 1:**
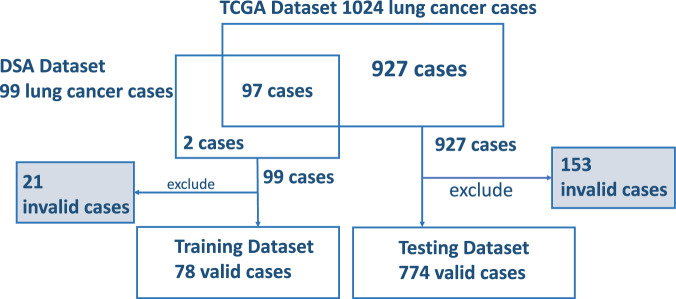
A representation of the number of reports at each stage of the process. Exclusions are accounted for due to reasons such as empty reports, poor scanning quality, and other factors, including reports of stage IV or unknown conditions.

From all the downloaded 99 pathology reports from CDSA for the training data, we excluded 21 invalid reports due to near-empty content, poor scanning quality, or missing report forms. Seventy-eight valid pathology reports were included as the training data to optimize the prompt. To evaluate the model performance, 1024 pathology reports were downloaded from cBioPortal. Among them, 97 overlapped with the training data and were excluded from the evaluation. We further excluded 153 invalid reports due to near-empty content, poor scanning quality, or missing report forms. The invalid reports were preserved to evaluate ChatGPT’s handling of irregular inputs separately, and were not included in the testing data for accuracy performance assessment. As a result, 774 valid pathology reports were included as the testing data for performance evaluation. These valid reports still contain typos, missing words, random characters, incomplete contents, and other quality issues challenging human reading. The corresponding numbers of reports used at each step of the process are detailed in Fig. 1.

The specific task of this study was to identify tumor staging and histology types which are important for clinical care and research from pathology reports. The TNM staging system^16^, outlining the primary tumor features (T), regional lymph node involvement (N), and distant metastases (M), is commonly used to define the disease extent, assign prognosis, and guide lung cancer treatment. The American Joint Committee on Cancer (AJCC) has periodically released various editions^16^ of TNM classification/staging for lung cancers based on recommendations from extensive database analyses. Following the AJCC guideline, individual pathologic T, N, and M stage components can be summarized into an overall pathologic staging score of Stage I, II, III, or IV. For this project, we instructed ChatGPT to use the AJCC 7^th^ edition Cancer Staging Manual^17^ as the reference for staging lung cancer cases. As the lung cancer cases in our dataset are predominantly non-metastatic, the pathologic metastasis (pM) stage was not extracted. The data elements we chose to extract and evaluate for this study are pathologic primary tumor (pT) and pathologic lymph node (pN) stage components, overall pathologic tumor stage, and histology type.

### Overall Performance

**Fig. 2:**
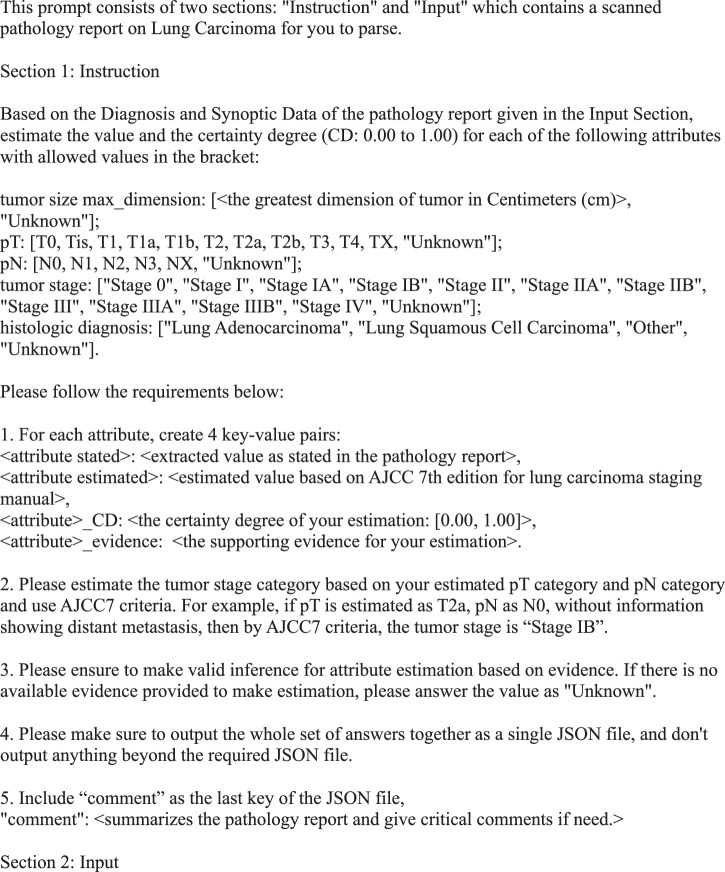
Final prompt for information extraction and estimation from pathology reports.

**Fig. 3:**
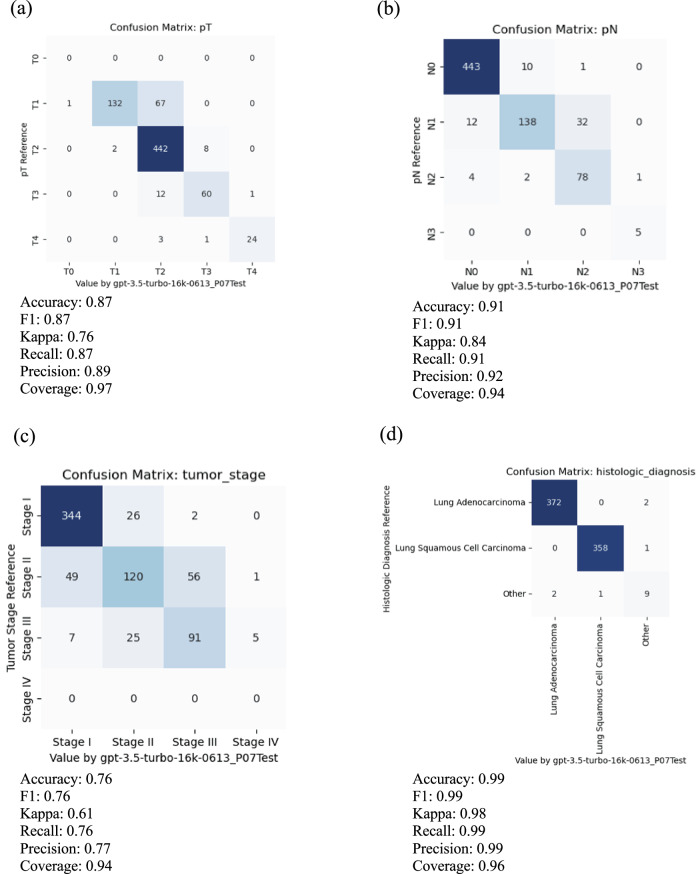
The performance of ChatGPT is measured by the confusion matrixes for the key attributes of interest on Test Data. For meaningful evaluation, the cases with uncertain values, such as “Not Available”, “Not Specified”, “Cannot be determined”, “Unknown”, et al. in reference and prediction have been removed. **a** Primary tumor features (pT), **b** regional lymph node involvement (pN), **c** overall tumor stage, and **d** histological diagnosis.

Using the training data in the CDSA dataset (*n* = 78), we experimented and improved prompts iteratively, and the final prompt is presented in Fig. 2. The overall performance of the ChatGPT (gpt-3.5-turbo-16k model) is evaluated in the TCGA dataset (*n* = 774), and the results are summarized in Table 1. The accuracy of primary tumor features (pT), regional lymph node involvement (pN), overall tumor stage, and histological diagnosis are 0.87, 0.91, 0.76, and 0.99, respectively. The average accuracy of all attributes is 0.89. The coverage rates for pT, pN, overall stage and histological diagnosis are 0.97, 0.94, 0.94 and 0.96, respectively. Further details of the accuracy evaluation, F1, Kappa, recall, and precision for each attribute are summarized as confusion matrices in Fig. 3.

**Table 1:** Overall performance of ChatGPT on data extraction from pathology reports

Attribute	Accuracy	F1	Kappa	Recall	Precision	Coverage
Primary tumor features (pT)	0.87	0.87	0.76	0.87	0.89	0.97
Regional lymph node involvement (pN)	0.91	0.91	0.84	0.91	0.92	0.94
Overall tumor stage	0.76	0.76	0.61	0.76	0.77	0.94
Histological diagnosis	0.99	0.99	0.98	0.99	0.99	0.96
Average	0.89	0.88	0.80	0.89	0.89	0.95

### Inference and Interpretation

**Fig. 4:**
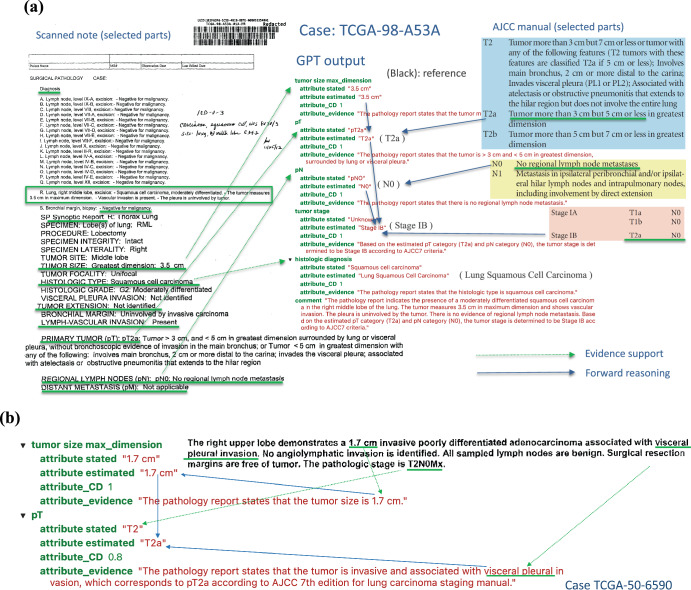
Case analysis to illustrate how ChatGPT reads the pathology report and makes inference. **a** TCGA-98-A53A. An example of a scanned pathological report (left panel) and ChatGPT output and interpretation (right panel). All estimations and support evidence are consistent with the pathologist’s evaluations. **b** The GPT model correctly inferred pT as T2a based on the tumor’s size and involvement according to AJCC guidelines.

To understand how ChatGPT reads and makes inferences from pathology reports, we demonstrated a case study using a typical pathology report in this cohort (TCGA-98-A53A) in Fig. 4a. The left panel shows part of the original pathology report, and the right panel shows the ChatGPT output with estimated pT, pN, overall stage, and histology diagnosis. For each estimate, ChatGPT gives the confidence level and the corresponding evidence it used for the estimation. In this case, ChatGPT correctly extracted information related to tumor size, tumor features, lymph node involvement, and histology information and used the AJCC staging guidelines to estimate tumor stage correctly. In addition, the confidence level, evidence interpretation, and case summary align well with the report and pathologists’ evaluations. For example, the evidence for the pT category was described as “The pathology report states that the tumor is > 3 cm and < 5 cm in greatest dimension, surrounded by lung or visceral pleura.” The evidence for tumor stage was described as “Based on the estimated pT category (T2a) and pN category (N0), the tumor stage is determined to be Stage IB according to AJCC7 criteria.” It shows that ChatGPT extracted relevant information from the note and correctly inferred the pT category based on the AJCC guideline (Supplementary Fig. 1) and the extracted information.

In another more complex case, TCGA-50-6590 (Fig. 4b), ChatGPT correctly inferred pT as T2a based on both the tumor’s size and location according to AJCC guidelines. Case TCGA-44-2656 demonstrates a more challenging scenario (Supplementary Fig. 2), where the report only contains some factual data without specifying pT, pN, and tumor stage. However, ChatGPT was able to infer the correct classifications based on the reported facts and provide proper supporting evidence.

### Error analysis

To understand the types and potential reasons for misclassifications, we performed a detailed error analysis by looking into individual attributes and cases where ChatGPT made mistakes, the results of which are summarized below.

#### Primary tumor feature (pT) classification

In total, 768 cases with valid reports and reference values in the testing data were used to evaluate the classification performance of pT. Among them, 15 cases were reported with unknown or empty output by ChatGPT, making the coverage rate 0.97. For the remaining 753 cases, 12.6% of pT was misclassified. Among these misclassification cases, the majority were T1 misclassified as T2 (67 out of 753 or 8.9%) or T3 misclassified as T2 (12 out of 753, or 1.6%).

**Fig. 5:**
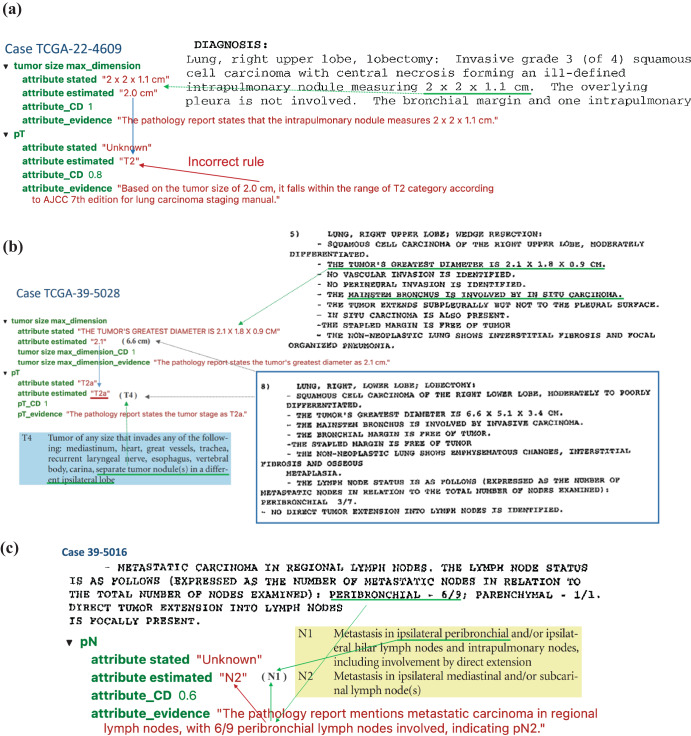
Case analysis to reveal typical errors in ChatGPT outputs. **a** TCGA-22-4609 illustrates a typical case where the GPT model uses a false rule, which is incorrect by AJCC guideline. **b** Case TCGA-39-5028 shows a complex case where there exist two tumors and the GPT model only capture one of them. **c** Case TCGA-39-5016 reveals a case where the GPT model made a mistake for getting confused with domain terminology.

In most cases, ChatGPT extracted the correct tumor size information but used an incorrect rule to distinguish pT categories. For example, in the case TCGA-22-4609 (Fig. 5a), ChatGPT stated, “Based on the tumor size of 2.0 cm, it falls within the range of T2 category according to AJCC 7th edition for lung carcinoma staging manual.” However, according to the AJCC 7^th^ edition staging guidelines for lung cancer, if the tumor is more than 2 cm but less than 3 cm in greatest dimension and does not invade nearby structures, pT should be classified as T1b. Therefore, ChatGPT correctly extracted the maximum tumor dimension of 2 cm but incorrectly interpreted this as meeting the criteria for classification as T2. Similarly, for case TCGA-85-A4JB, ChatGPT incorrectly claimed, “Based on the tumor size of 10 cm, the estimated pT category is T2 according to AJCC 7th edition for lung carcinoma staging manual.” According to the AJCC 7^th^ edition staging guidelines, a tumor more than 7 cm in greatest dimension should be classified as T3.

Another challenging situation arose when multiple tumor nodules were identified within the lung. In the case of TCGA-39-5028 (Fig. 5b), two separate tumor nodules were identified: one in the right upper lobe measuring 2.1 cm in greatest dimension and one in the right lower lobe measuring 6.6 cm in greatest dimension. According to the AJCC 7^th^ edition guidelines, the presence of separate tumor nodules in a different ipsilateral lobe results in a classification of T4. However, ChatGPT classified this case as T2a, stating, “The pathology report states the tumor’s greatest diameter as 2.1 cm”. This classification would be appropriated if the right upper lobe nodule were a single isolated tumor. However, ChatGPT failed to consider the presence of the second, larger nodule in the right lower lobe when determining the pT classification.

#### Regional lymph node involvement (pN)

The classification performance of pN was evaluated using 753 cases with valid reports and reference values in the testing data. Among them, 27 cases were reported with unknown or empty output by ChatGPT, making the coverage rate 0.94. For the remaining 726 cases, 8.5% of pN was misclassified. Most of these misclassification cases were N1 misclassified as N2 (32 cases). The AJCC 7th edition staging guidelines use the anatomic locations of positive lymph nodes to determine N1 vs. N2. However, most of the misclassification cases were caused by ChatGPT interpreting the number of positive nodes rather than the locations of the positive nodes. One such example is the case TCGA-85-6798. The report states, “Lymph nodes: 2/16 positive for metastasis (Hilar 2/16)”. Positive hilar lymph nodes correspond to N1 classification according to AJCC 7th edition guidelines. However, ChatGPT misclassifies this case as N2, stating, “The pathology report states that 2 out of 16 lymph nodes are positive for metastasis. Based on this information, the pN category can be estimated as N2 according to AJCC 7th edition for lung carcinoma staging manual.” This interpretation is incorrect, as the number of positive lymph nodes is not part of the criteria used to determine pN status according to AJCC 7th edition guidelines. The model misinterpreted pN2 predictions in 22 cases due to similar false assertions.

In some cases, the ChatGPT model made classification mistakes by misunderstanding the locations’ terminology. Figure 5c shows a case (TCGA-39-5016) where the ChatGPT model recognized that “6/9 peribronchial lymph nodes involved, “ corresponding with classification as N1, but ChatGPT misclassified this case as N2. By AJCC 7th edition guidelines, N2 is defined as “Metastasis in ipsilateral mediastinal and/or subcarinal lymph node(s)”. The ChatGPT model did not fully understand that terminology and made misclassifications.

#### Pathology tumor stage

**Fig. 6:**
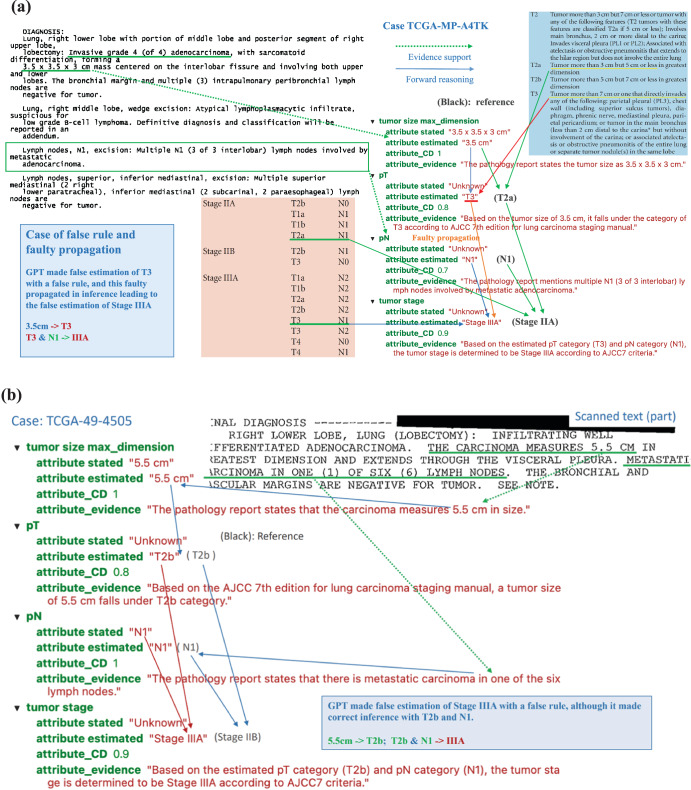
Case analysis to reveal more error scenarios. **a** Case TCGA-MP-A4TK: An example of typical errors GPT made in the experiments, i.e. GPT took false rule and further led to faulty propagation. **b** Case TCGA-49-4505: The GPT model made false estimation of Stage IIIA with a false rule, although it made correct inference with T2b and N1.

The overall tumor stage classification performance was evaluated using 744 cases with valid reports and reference values as stage I, II and III in the testing data. Among them, 18 cases were reported as unknown or empty output by ChatGPT making the coverage rate as 0.94. For the remaining 726 cases, 23.6% of the overall stage was misclassified. Since the overall stage depends on individual pT and pN stages, the mistakes could come from misclassification of pT or pN (error propagation) or applying incorrect inference rules to determine the overall stage from pT and pN (incorrect rules). Looking into the 56 cases where ChatGPT misclassified stage II as stage III, 22 cases were due to error propagation, and 34 were due to incorrect rules. Figure 6a shows an example of error propagation (TCGA-MP-A4TK). ChatGPT misclassified the pT stage from T2a to T3, and then this mistake led to the incorrect classification of stage IIA to stage IIIA. Figure 6b illustrates a case (TCGA-49-4505) where ChatGPT made correct estimation of pT and pN but made false prediction about tumor stage by using a false rule. Among the 34 cases affected by incorrect rules, ChatGPT mistakenly inferred tumor stage as stage III for 26 cases where pT is T3 and pN is N0, respectively. For example, for case TCGA-55-7994, ChatGPT provided the evidence as “Based on the estimated pT category (T3) and pN category (N0), the tumor stage is determined to be Stage IIIA according to AJCC7 criteria”. According to AJCC7, tumors with T3 and N0 should be classified as stage IIB. Similarly, error analysis for other tumor stages shows that misclassifications come from both error propagation and applying false rules.

#### Histological diagnosis

The classification performance of histology diagnosis was evaluated using 762 cases with valid reports and reference values in the testing data. Among them, 17 cases were reported as either unknown or empty output by ChatGPT, making the coverage rate 0.96. For the remaining 745 cases, 6 ( < 1%) of histology types were misclassified. Among the mistakes that ChatGPT made for histology diagnosis, ChatGPT misclassified 3 of them as “other” type and 3 cases of actual “other” type (neither adenocarcinomas nor squamous cell carcinomas) as 2 adenocarcinomas and 1 squamous cell carcinoma. In TCGA-22-5485, two tumors exist: one squamous cell carcinoma and another adenocarcinoma, which should be classified as the ‘other’ type. However, ChatGPT only identified and extracted information for one tumor. In the case TCGA-33-AASB, which is the “other” type of histology, ChatGPT captured the key information and gave it as evidence: “The pathology report states the histologic diagnosis as infiltrating poorly differentiated non-small cell carcinoma with both squamous and glandular features”. However, it mistakenly estimated this case as “adenocarcinoma”. In another case (TCGA-86-8668) of adenocarcinoma, ChatGPT again captured key information and stated as evidence, “The pathology report states the histologic diagnosis as Bronchiolo-alveolar carcinoma, mucinous” but could not tell it is a subtype of adenocarcinoma. Both cases reveal that ChatGPT still has limitations in the specific domain knowledge in lung cancer pathology and the capability of correcting understanding its terminology.

### Analyzing irregularities

The initial model evaluation and prompt-response review uncovered irregular scenarios: the original pathology reports may be blank, poorly scanned, or simply missing report forms. We reviewed how ChatGPT responded to these anomalies. First, when a report was blank, the prompt contained only the instruction part. ChatGPT failed to recognize this situation in most cases and inappropriately generated a fabricated case. Our experiments showed that, with the temperature set at 0 for blank reports, ChatGPT converged to a consistent, hallucinated response. Second, for nearly blank reports with a few random characters and poorly scanned reports, ChatGPT consistently converged to the same response with increased variance as noise increased. In some cases, ChatGPT responded appropriately to all required attributes but with unknown values for missing information. Last, among the 15 missing report forms in a small dataset, ChatGPT responded “unknown” as expected in only 5 cases, with the remaining 10 still converging to the hallucinated response.

### Reproducibility evaluation

Since ChatGPT models (even with the same version) evolve over time, it is important to evaluate the stability and reproducibility of ChatGPT. For this purpose, we conducted experiments with the same model (“gpt-3.5-turbo-0301”), the same data, prompt, and settings (e.g., temperature = 0) twice in early April and the middle of May of 2023. The rate of equivalence between ChatGPT estimations in April and May on key attributes of interest (pT, pN, tumor stage, and histological diagnosis) is 0.913. The mean absolute error between certainty degrees in the two experiments is 0.051. Considering the evolutionary nature of ChatGPT models, we regard an output difference to a certain extent as reasonable and the overall ChatGPT 3.5 model as stable.

### Comparison with other NLP methods

In order to have a clear perspective on how ChatGPT’s performance stands relative to established methods, we conducted a comparative analysis of the results generated by ChatGPT with two established methods: a keyword search algorithm and a deep learning-based Named Entity Recognition (NER) method.

#### Data selection and annotation

Since the keyword search and NER methods do not support zero-shot learning and require human annotations on the entity level, we carefully annotated our dataset for these traditional NLP methods. We used the same training and testing datasets as in the prompt engineering for ChatGPT. The training dataset underwent meticulous annotation by experienced medical professionals, adhering to the AJCC7 standards. This annotation process involved identifying and highlighting all relevant entities and text spans related to stage, histology, pN, and pT attributes. The detailed annotation process for the 78 cases required a few weeks of full-time work from medical professionals.

#### Keyword search algorithm using wordpiece tokenizer

For the keyword search algorithm, we employed the WordPiece tokenizer to segment words into subwords. We compiled an annotated entity dictionary from the training dataset. To assess the performance of this method, we calculated span similarities between the extracted spans in the validation and testing datasets and the entries in the dictionary.

#### Named Entity Recognition (NER) classification algorithm

For the NER classification algorithm, we designed a multi-label span classification model. This model utilized the pre-trained Bio_ClinicalBERT as its backbone. To adapt it for multi-label classification, we introduced an additional linear layer. The model underwent fine-tuning for 1000 epochs using the stochastic gradient descent (SGD) optimizer. The model exhibiting the highest overall F1 score on the validation dataset was selected as the final model for further evaluation in the testing dataset.

#### Performance evaluation

We evaluated the performance of both the keyword search and NER methods on the testing dataset. We summarized the predicted entities/spans and their corresponding labels. In cases where multiple related entities were identified for a specific category, we selected the most severe entities as the final prediction. Moreover, we inferred the stage information for corpora lacking explicit staging information by aggregating details from pN, pT, and diagnosis, aligning with the AJCC7 protocol. The overall predictions for stage, diagnosis, pN, and pT were compared against the ground truth table to gauge the accuracy and effectiveness of our methods. The results (Supplementary Table S1) show that the ChatGPT outperforms WordPiece tokenizer and NER Classifier. The average accuracy for ChatGPT, WordPiece tokenizer, and NER Classifier are 0.89, 0.51, and 0.76, respectively.

### Prompt engineering process and results

Prompt design is a heuristic search process with many elements to consider, thus having a significantly large design space. We conducted many experiments to explore better prompts. Here, we share a few typical prompts and the performance of these prompts in the training data set to demonstrate our prompt engineering process.

#### Output format

The most straightforward prompt without special design would be: “read the pathology report and answer what are pT, pN, tumor stage, and histological diagnosis”. However, this simple prompt would make ChatGPT produce unstructured answers varying in format, terminology, and granularity across the large number of pathology reports. For example, ChatGPT may output pT as “T2” or “pT2NOMx”, and it outputs histological diagnosis as “Multifocal invasive moderately differentiated non-keratinizing squamous cell carcinoma”. The free-text answers will require a significant human workload to clean and process the output from ChatGPT. To solve this problem, we used a multiple choice answer format to force ChatGPT to pick standardized values for some attributes. For example, for pT, ChatGPT could only provide the following outputs: “T0, Tis, T1, T1a, T1b, T2, T2a, T2b, T3, T4, TX, Unknown”. For the histologic diagnosis, ChatGPT could provide output in one of these categories: Lung Adenocarcinoma, Lung Squamous Cell Carcinoma, Other, Unknown. In addition, we added the instruction, “Please make sure to output the whole set of answers together as a single JSON file, and don’t output anything beyond the required JSON file,” to emphasize the requirement for the output format. These requests in the prompt make the downstream analysis of ChatGPT output much more efficient. In order to know the certainty degree of ChatGPT’s estimate and the evidence, we asked ChatGPT to provide the following 4 outputs for each attribute/variable: extracted value as stated in the pathology report, estimated value based on AJCC 7th edition for lung carcinoma staging manual, the certainty degree of the estimation, and the supporting evidence for the estimation. The classification accuracy of this prompt with multiple choice output format (prompt v1) in our training data could achieve 0.854.

#### Evidence-based inference

One of the major concerns for LLM is that the results from the model are not supported by any evidence, especially when there is not enough information for specific questions. In order to reduce this problem, we emphasize the use of evidence for inference in the prompt by adding this instruction to ChatGPT: “Please ensure to make valid inferences for attribute estimation based on evidence. If there is no available evidence provided to make an estimation, please answer the value as “Unknown.” In addition, we asked ChatGPT to “Include “comment” as the last key of the JSON file.” After adding these two instructions (prompt v2), the performance of the classification in the training data increased to 0.865.

#### Chain of thought prompting by asking intermediate questions

Although tumor size is not a primary interest for diagnosis and clinical research, it plays a critical role in classifying the pT stage. We hypothesize that if ChatGPT pays closer attention to tumor size, it will have better classification performance. Therefore, we added an instruction in the prompt (prompt v3) to ask ChatGPT to estimate: “tumor size max_dimension: [<the greatest dimension of tumor in Centimeters (cm)>, ‘Unknown’]” as one of the attributes. After this modification, the performance of the classification in the training data increased to 0.90.

#### Providing examples

Providing examples is an effective way for humans to learn, and it should have similar effects for ChatGPT. We provided a specific example to infer the overall stage based on pT and pN by adding this instruction: “Please estimate the tumor stage category based on your estimated pT category and pN category and use AJCC7 criteria. For example, if pT is estimated as T2a and pN as N0, without information showing distant metastasis, then by AJCC7 criteria, the tumor stage is “Stage IB”.” After this modification (prompt v4), the performance of the classification in the training data increased to 0.936.

Although we can further refine and improve prompts, we decided to use prompt v4 as the final model and apply it to the testing data and get the final classification accuracy of 0.89 in the testing data.

### ChatGPT-4 performance

LLM evolves rapidly and OpenAI just released the newest GPT-4 Turbo model (GPT-4-1106-preview) in November 2023. To compare this new model with GPT-3.5-Turbo, we applied this newest GPT model GPT-4-1106 to analyze all the lung cancer pathology notes in the testing data. The classification result and the comparison with the GPT-3.5-Turbo-16k are summarized in Supplementary Table 1. The results show that GPT-4-turbo performs better in almost every aspect; overall, the GPT-4-turbo model increases performance by over 5%. However, GPT-4-Turbo is much more expensive than GPT-3.5-Turbo. The performance of GPT-3.5-Turbo-16k is still comparable and acceptable. As such, this study mainly focuses on assessing GPT-3.5-Turbo-16k, but highlights the fast development and promise of using LLM to extract structured data from clinical notes.

### Analyzing osteosarcoma data

To demonstrate the broader application of this method beyond lung cancer, we collected and analyzed clinical notes from pediatric osteosarcoma patients. Osteosarcoma, the most common type of bone cancer in children and adolescents, has seen no substantial improvement in patient outcomes for the past few decades^18^. Histology grades and margin status are among the most important prognostic factors for osteosarcoma. We collected pathology reports from 191 osteosarcoma cases (approved by UTSW IRB #STU 012018-061). Out of these, 148 cases had histology grade information, and 81 had margin status information; these cases were used to evaluate the performance of the GPT-3.5-Turbo-16K model and our prompt engineering strategy. Final diagnoses on grade and margin were manually reviewed and curated by human experts, and these diagnoses were used to assess ChatGPT’s performance. All notes were de-identified prior to analysis. We applied the same prompt engineering strategy to extract grade and margin information from these osteosarcoma pathology reports. This analysis was conducted on our institution’s private Azure OpenAI platform, using the GPT-3.5-Turbo-16K model (version 0613), the same model used for lung cancer cases. ChatGPT accurately classified both grades (with a 98.6% accuracy rate) and margin status (100% accuracy), as shown in Supplementary Fig. 3. In addition, Supplementary Fig. 4 details a specific case, illustrating how ChatGPT identifies grades and margin status from osteosarcoma pathology reports.

## Discussion

Since ChatGPT’s release in November 2022, it has spurred many potential innovative applications in healthcare^19–23^. To our knowledge, this is among the first reports of an end-to-end data science workflow for prompt engineering, using, and rigorously evaluating ChatGPT in its capacity of batch-processing information extraction tasks on large-scale clinical report data.

The main obstacle to developing traditional medical NLP algorithms is the limited availability of annotated data and the costs for new human annotations. To overcome these hurdles, particularly in integrating problem-specific information and domain knowledge with LLMs’ task-agnostic general knowledge, Augmented Language Models (ALMs)^24^, which incorporate reasoning and external tools for interaction with the environment, are emerging. Research shows that in-context learning (most influentially, few-shot prompting) can complement LLMs with task-specific knowledge to perform downstream tasks effectively^24,25^. In-context learning is an approach of training through instruction or light tutorial with a few examples (so called few-shot prompting; well instruction without any example is called 0-shot prompting) rather than fine-tuning or computing-intensive training, which adjusts model weights. This approach has become a dominant method for using LLMs in real-world problem-solving^24–26^. The advent of ALMs promises to revolutionize almost every aspect of human society, including the medical and healthcare domains, altering how we live, work, and communicate. Our study shows the feasibility of using ChatGPT to extract data from free text without extensive task-specific human annotation and model training.

In medical data extraction, our study has demonstrated the advantages of adopting ChatGPT over traditional methods in terms of cost-effectiveness and efficiency. Traditional approaches often require labor-intensive annotation processes that may take weeks and months from medical professionals, while ChatGPT models can be fine-tuned for data extraction within days, significantly reducing the time investment required for implementation. Moreover, our economic analysis revealed the cost savings associated with using ChatGPT, with processing over 900 pathology reports incurring a minimal monetary cost (less than $10 using GPT 3.5 Turbo and less than $30 using GPT-4 Turbo). This finding underscores the potential benefits of incorporating ChatGPT into medical data extraction workflows, not only for its time efficiency but also for its cost-effectiveness, making it a compelling option for medical institutions and researchers seeking to streamline their data extraction processes without compromising accuracy or quality.

A critical requirement for effectively utilizing an LLM is crafting a high-quality “prompt” to instruct the LLM, which has led to the emergence of an important methodology referred to as “prompt engineering.” Two fundamental principles guide this process: firstly, the provision of appropriate context, and secondly, delivering clear instructions about subtasks and the requirements for the desired response and how it should be presented. For a single query for one-time use, the user can experiment with and revise the prompt within the conversation session until a satisfactory answer is obtained. However, prompt design can become more complex when handling repetitive tasks over many input data files using the OpenAI API. In these instances, a prompt must be designed according to a given data feed while maintaining the generality and coverage for various input data features. In this study, we found that providing clear guidance on the output format, emphasizing evidence-based inference, providing chain of thought prompting by asking for tumor size information, and providing specific examples are critical in improving the efficiency and accuracy of extracting structured data from the free-text pathology reports. The approach employed in this study effectively leverages the OpenAI API for batch queries of ChatGPT services across a large set of tasks with similar input data structures, including but not limited to pathology reports and EHR.

Our evaluation results show that the ChatGPT (gpt-3.5-turbo-16k) achieved an overall average accuracy of 89% in extracting and estimating lung cancer staging information and histology subtypes compared to pathologist-curated data. This performance is very promising because some scanned pathology reports included in this study contained random characters, missing parts, typos, varied formats, and divergent information sections. ChatGPT also outperformed traditional NLP methods. Our case analysis shows that most misclassifications were due to a lack of knowledge of detailed pathology terminology or very specialized information in the current versions of ChatGPT models, which could be avoided with future model training or fine-tuning with more domain-specific knowledge.

While our experiments reveal ChatGPT’s strengths, they also underscore its limitations and potential risks, the most significant being the occasional “hallucination” phenomenon^27,28^, where the generated content is not faithful to the provided source content. For example, the responses to blank or near-blank reports reflect this issue, though these instances can be detected and corrected due to convergence towards an “attractor”.

The phenomenon of ‘hallucination’ in LLMs presents a significant challenge in the field. It is important to consider several key factors to effectively address the challenges and risks associated with ChatGPT’s application in medicine. Since the output of an LLM depends on both the model and the prompt, mitigating hallucination can be achieved through improvements in GPT models and prompting strategies. From a model perspective, model architecture, robust training, and fine-tuning on a diverse and comprehensive medical dataset, emphasizing accurate labeling and classification, can reduce misclassifications. Additionally, enhancing LLMs’ comprehension of medical terminology and guidelines by incorporating feedback from healthcare professionals during training and through Reinforcement Learning from Human Feedback (RLHF) can further diminish hallucinations. Regarding prompt engineering strategies, a crucial method is to prompt the GPT model with a ‘chain of thought’ and request an explanation with the evidence used in the reasoning. Further improvements could include explicitly requesting evidence from input data (e.g., the pathology report) and inference rules (e.g., AJCC rules). Prompting GPT models to respond with ‘Unknown’ when information is insufficient for making assertions, providing relevant context in the prompt, or using ‘embedding’ of relevant text to narrow down the semantic subspace can also be effective. Harnessing hallucination is an ongoing challenge in AI research, with various methods being explored^5,27^. For example, a recent study proposed “SelfCheckGPT” approach to fact-check black-box models^29^. Developing real-time error detection mechanisms is crucial for enhancing the reliability and trustworthiness of AI models. More research is needed to evaluate the extent, impacts, and potential solutions of using LLMs in clinical research and care.

When considering using ChatGPT and similar LLMs in healthcare, it’s important to thoughtfully consider the privacy implications. The sensitivity of medical data, governed by rigorous regulations like HIPAA, naturally raises concerns when integrating technologies like LLMs. Although it is a less concern to analyze public available de-identified data, like the lung cancer pathology notes used in this study, careful considerations are needed for secured healthcare data. More secured OpenAI services are offered by OpenAI security portal, claimed to be compliant to multiple regulation standards, and Microsoft Azure OpenAI, claimed could be used in a HIPAA-compliant manner. For example, de-identified Osteosarcoma pathology notes were analyzed by Microsoft Azure OpenAI covered by the Business Associate Agreement in this study. In addition, exploring options such as private versions of these APIs, or even developing LLMs within a secure healthcare IT environment, might offer good alternatives. Moreover, implementing strong data anonymization protocols and conducting regular security checks could further protect patient information. As we navigate these advancements, it’s crucial to continuously reassess and adapt appropriate privacy strategies, ensuring that the integration of AI into healthcare is both beneficial and responsible.

Despite these challenges, this study demonstrates our effective methodology in “prompt engineering”. It presents a general framework for using ChatGPT’s API in batch queries to process large volumes of pathology reports for structured information extraction and estimation. The application of ChatGPT in interpreting clinical notes holds substantial promise in transforming how healthcare professionals and patients utilize these crucial documents. By generating concise, accurate, and comprehensible summaries, ChatGPT could significantly enhance the effectiveness and efficiency of extracting structured information from unstructured clinical texts, ultimately leading to more efficient clinical research and improved patient care.

In conclusion, ChatGPT and other LLMs are powerful tools, not just for pathology report processing but also for the broader digital transformation of healthcare documents. These models can catalyze the utilization of the rich historical archives of medical practice, thereby creating robust resources for future research.

## Methods

### Data processing, workflow, and prompt engineering

The lung cancer data we used for this study are publicly accessible via CDSA (https://cancer.digitalslidearchive.org/) and TCGA (https://cBioPortal.org), and they are de-identified data. The institutional review board at the University of Texas Southwestern Medical Center has approved this study where patient consent was waived for using retrospective, de-identified electronic health record data.

**Fig. 7:**
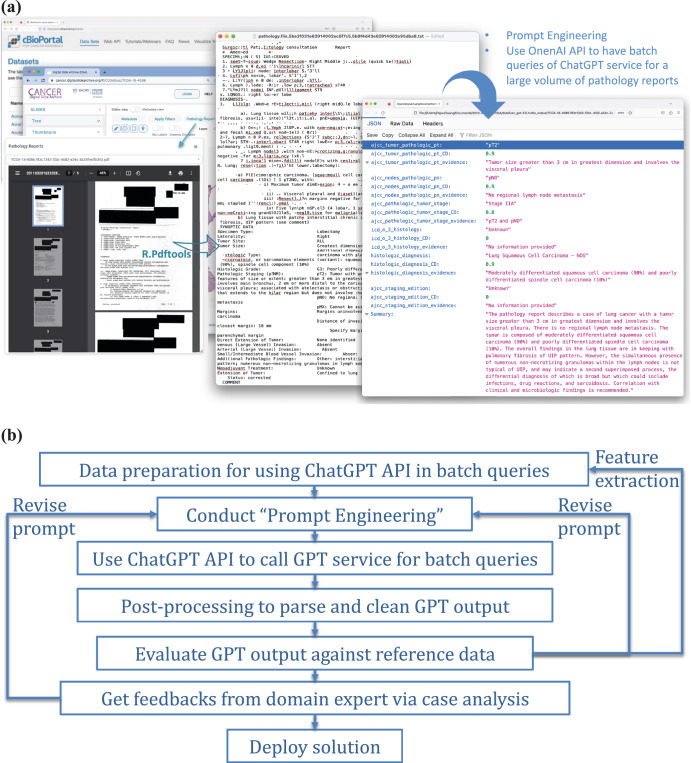
An overview of the process and framework of using ChatGPT for structured data extraction from pathology reports. **a** Illustration of the use of OpenAI API for batch queries of ChatGPT service, applied to a substantial volume of clinical notes — pathology reports in our study. **b** A general framework for integrating ChatGPT into real-world applications.

We aimed to leverage ChatGPT to extract and estimate structured data from these notes. Figure 7a displays our process. First, scanned pathology reports in PDF format were downloaded from TCGA and CDSA databases. Second, R package pdftools, an optical character recognition tool, was employed to convert scanned PDF files into text format. After this conversion, we identified reports with near-empty content, poor scanning quality, or missing report forms, and those cases were excluded from the study. Third, the OpenAI API was used to analyze the text data and extract structured data elements based on specific prompts. In addition, we extracted case identifiers and metadata items from the TCGA metadata file, which was used to evaluate the model performance.

In this study, we implemented a problem-solving framework rooted in data science workflow and systems engineering principles, as depicted in Fig. 7b. An important step is the spiral approach^30^ to ‘prompt engineering’, which involves experimenting with subtasks, different phrasings, contexts, format specifications, and example outputs to improve the quality and relevance of the model’s responses. It was an iterative process to achieve the desired results. For the prompt engineering, we first define the objective: to extract information on TNM staging and histology type as structured attributes from the unstructured pathology reports. Second, we assigned specific tasks to ChatGPT, including estimating the targeted attributes, evaluating certainty levels, identifying key evidence of each attribute estimation, and generating a summary as output. The output was compiled into a JSON file. In this process, clinicians were actively formulating questions and evaluating the results.

Our study used the “gpt-3.5-turbo” model, accessible via the OpenAI API. The model incorporates 175 billion parameters and was trained on various public and authorized documents, demonstrating specific Artificial General Intelligence (AGI) capabilities^5^. Each of our queries sent to ChatGPT service is a “text completion”^31^, which can be implemented as a single round chat completion. All LLMs have limited context windows, constraining the input length of a query. Therefore, lengthy pathology reports combined with the prompt and ChatGPT’s response might exceed this limit. We used OpenAI’s “tiktoken” Python library to estimate the token count to ensure compliance. This constraint has been largely relaxed by the newly released GPT models with much larger context windows. We illustrate the pseudocode for batch ChatGPT queries on a large pathology report set in Supplementary Fig. 5.

### Model evaluation

We evaluated the performance of ChatGPT by comparing its output with expert-curated data elements provided in the TCGA structured data using the testing data set. Some staging records in the TCGA structured data needed to be updated; our physicians curated and updated those records. To mimic a real-world setting, we processed all reports regardless of data quality to collect model responses. For performance evaluation, we only used valid reports providing meaningful text and excluded the reports with near-empty content, poor scanning quality, and missing report forms, which were reported as irregular cases. We assessed the classification accuracy, F1, Kappa, recall, and precision for each attribute of interest, including pT, pN, overall stage, and histology types, and presented results as accuracy and confusion matrices. Missing data were excluded from the accuracy evaluation, and the coverage rate was reported for predicted values as ‘unknown’ or empty output.

### Reporting summary

Further information on research design is available in the Nature Research Reporting Summary linked to this article.

### Supplementary information

Supplemental Figures and Tables

Reporting Summary

## Data Availability

The lung cancer dataset we used for this study is “Pan-Lung Cancer (TCGA, Nat Genet2016)”, (https://www.cbioportal.org/study/summary?id=nsclc_tcga_broad_2016) and the “luad” and “lusc” subsets from CDSA (https://cancer.digitalslidearchive.org/). We have provided a reference regarding how to access the data^32^. We utilized the provided APIs to retrieve clinical information and pathology reports for the LUAD (lung adenocarcinoma) and LUSC (lung squamous cell carcinoma) cohorts. The pediatric data are the EHR data from UTSW clinic services. The data is available from the corresponding author upon reasonable request and IRB approval.

## Code availability

All codes used in this paper were developed using APIs from OpenAI. The prompt for the API is available in Fig. 2. Method-specific code is available from the corresponding author upon request.
