# Supplementary material for: A critical assessment of using ChatGPT for extracting structured data from clinical notes
Source: NPJ Digit Med. 2024 May 1;7:106. doi: 10.1038/s41746-024-01079-8 (PMC11063058; doi:10.1038/s41746-024-01079-8)
Supplement: Supplementary file 1 — Supplemental Figures and Tables [file 41746_2024_1079_MOESM1_ESM.pdf]

T (Primary Tumor)	
TX	Primary tumor cannot be assessed, or tumor proven by the presence of malignant cells in sputum or bronchial washings but not visualized by imaging or bronchoscopy
T0	No evidence of primary tumor
Tis	Carcinoma in situ
T1	Tumor ≤3 cm in greatest dimension, surrounded by lung or visceral pleura, without bronchoscopic evidence of invasion more proximal than the lobar bronchus (i.e., not in the main bronchus)[a]
T1a	Tumor ≤2 cm in greatest dimension
T1b	Tumor >2 cm but ≤3 cm in greatest dimension
T2	Tumor >3 cm but ≤7 cm or tumor with any of the following features (T2 tumors with these features are classified T2a if ≤5 cm)
	Involves main bronchus, ≥2 cm distal to the carina
	Invades visceral pleura
	Associated with atelectasis or obstructive pneumonitis that extends to the hilar region but does not involve the entire lung
T2a	Tumor >3 cm but ≤5 cm in greatest dimension
T2b	Tumor >5 cm but ≤7 cm in greatest dimension
T3	Tumor >7 cm or one that directly invades any of the following: chest wall (including superior sulcus tumors), diaphragm, phrenic nerve, mediastinal pleura, parietal pericardium; or tumor in the main bronchus <2 cm distal to the carina[a] but without involvement of the carina; or associated atelectasis or obstructive pneumonitis of the entire lung or separate tumor nodule(s) in the same lobe
T4	Tumor of any size that invades any of the following: mediastinum, heart, great vessels, trachea, recurrent laryngeal nerve, esophagus, vertebral body, carina; separate tumor nodule(s) in a different ipsilateral lobe

N (Regional Lymph Nodes)	
NX	Regional lymph nodes cannot be assessed
N0	No regional lymph node metastasis
N1	Metastasis in ipsilateral peribronchial and/or ipsilateral hilar lymph nodes and intrapulmonary nodes, including involvement by direct extension
N2	Metastasis in ipsilateral mediastinal and/or subcarinal lymph node(s)
N3	Metastasis in contralateral mediastinal, contralateral hilar, ipsilateral or contralateral scalene, or supraclavicular lymph node(s)
**M (Distant Metastasis)**	
MX	Distant metastasis cannot be assessed
M0	No distant metastasis
M1	Distant metastasis
M1a	Separate tumor nodule(s) in a contralateral lobe; tumor with pleural nodules or malignant pleural (or pericardial) effusion[b]
M1b	Distant metastasis

TABLE 4. Descriptors, Proposed T and M Categories, and Proposed Stage Groupings

Sixth Edition T/M Descriptor	Proposed T/M	N0	N1	N2	N3
T1 (≤2 cm)	T1a	IA	IIA	IIIA	IIIB
T1 (>2–3 cm)	T1b	IA	IIA	IIIA	IIIB
T2 (≤5 cm)	T2a	IB	**IIA**	IIIA	IIIB
T2 (>5–7 cm)	T2b	**IIA**	IIB	IIIA	IIIB
T2 (>7 cm)	T3	**IIB**	**IIIA**	IIIA	IIIB
T3 invasion		IIB	IIIA	IIIA	IIIB
T4 (same lobe nodules)		**IIB**	**IIIA**	**IIIA**	IIIB
T4 (extension)	T4	**IIIA**	**IIIA**	IIIB	IIIB
M1 (ipsilateral lung)		**IIIA**	**IIIA**	**IIIB**	**IIIB**
T4 (pleural effusion)	M1a	**IV**	**IV**	**IV**	**IV**
M1 (contralateral lung)		IV	IV	IV	IV
M1 (distant)	M1b	IV	IV	IV	IV

Cells in bold indicate a change from the sixth edition for a particular TNM category.

Supplementary Figure 1. AJCC TNM Staging Guide (7th Ed. From (*P. Goldstraw, et. al. 2007*)).

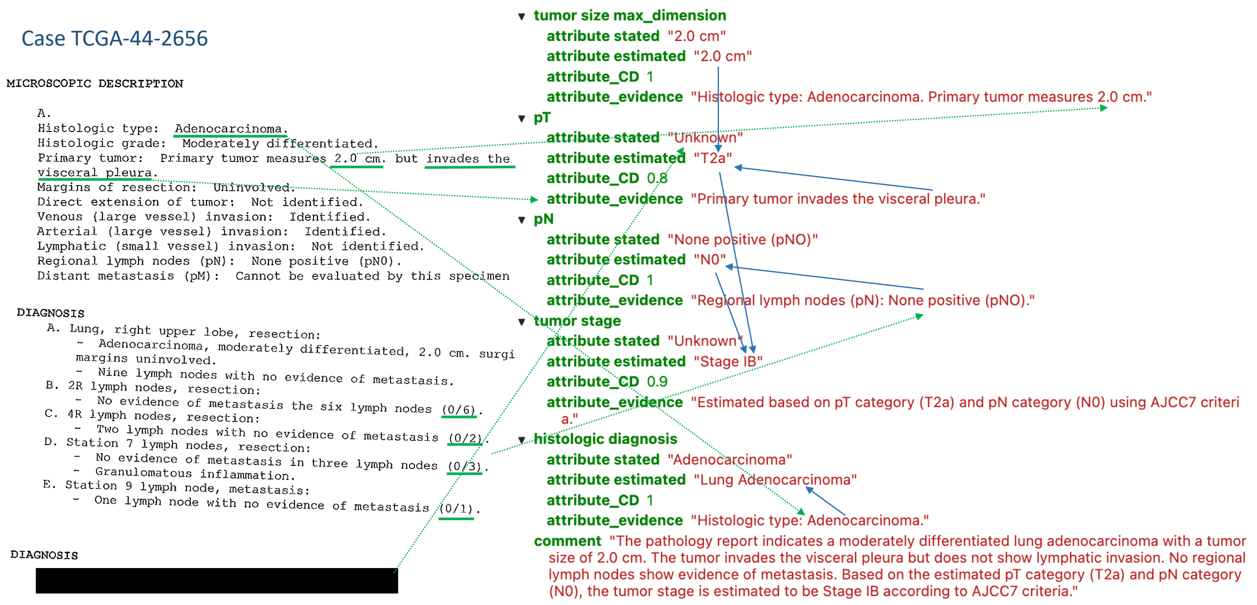

Supplementary Figure 2. Case TCGA-44-2656 shows that ChatGPT correctly extracted relevant information from the note and made sound inference about the pT category based on the AJCC guideline.

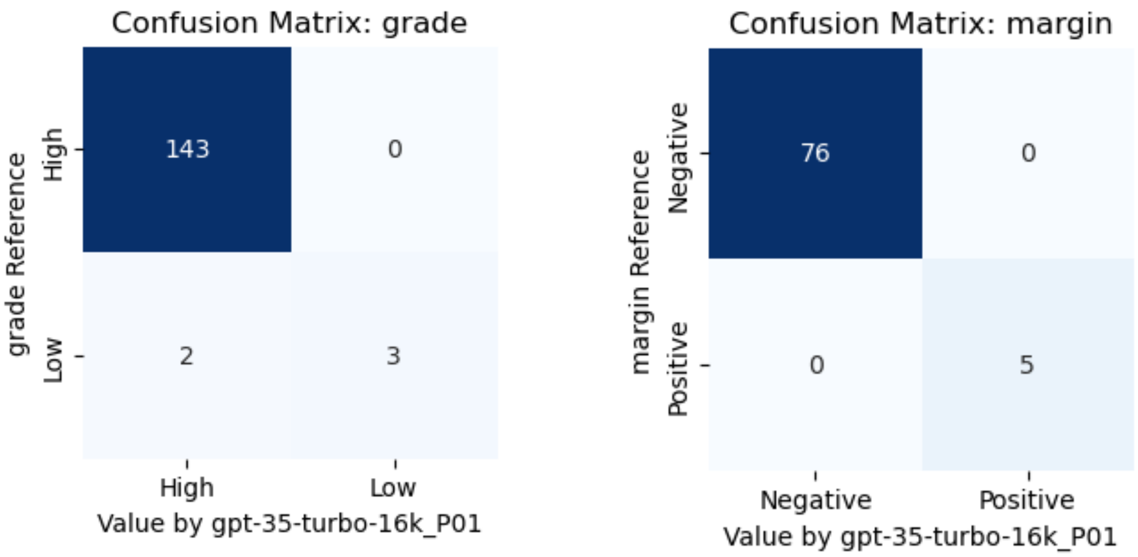

Supplementary Figure 3. The presented prompting strategy is applied structured data extraction from osteosarcoma clinic notes. The GPT model used for our experiment is GPT-3.5-Turbo-16K (version 0613) and it was deployed on dedicated AzureAI cloud service.

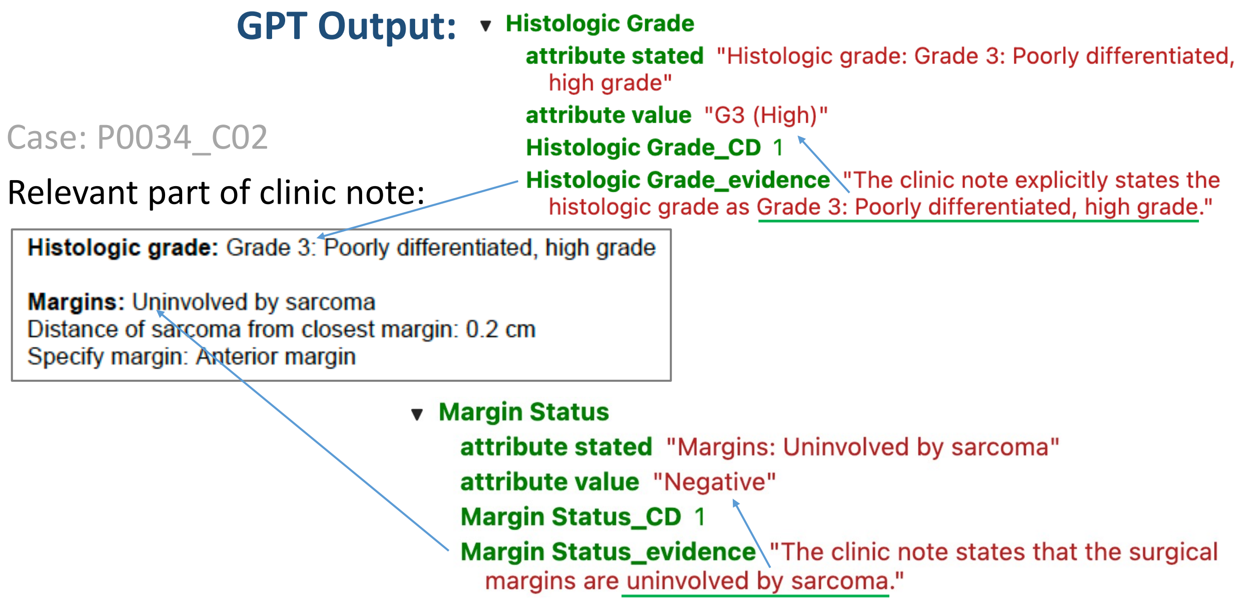

Supplementary Figure 4. An example to illustrate that the GPT model correctly captured histologic grade and margin category as well as the key evidence.

```
authenticate OpenAI API key
for each file in pathology report folder:
    read in report
    create instructive prompt for the report
    estimate number of tokens of the prompt with tiktokens
    if #tokens less than a threshold:
        try:
            construct chat message
            create a ChatCompletion with a GPT model and the message
            call OpenAI API and get the response
            extract the GPT model output
            write the GPT output to a file in GPT Output folder
        except:
            output message: the file is failed to get response
            append the file to unprocessed filelist
    else:
        append the file to unprocessed filelist
```

Supplementary Figure 5. Algorithm of GPT-Powered Pathology Report Reader

Supplementary Table 1. Performance of ChatGPT, WordPiece tokenizer and NER Classifier in terms of pN, pT, overall tumor stage and histological diagnosis

**A. primary tumor features (pT)**	**Accuracy**	**F1**	**Kappa**	**Recall**	**Precision**
GPT-3.5-Turbo-16K	0.87	0.87	0.76	0.87	0.89
GPT-4-Turbo-1106-preview	0.91	0.91	0.84	0.91	0.92
NER Classifier	0.35	0.42	0.14	0.35	0.65
WordPiece tokenizer	0.74	0.75	0.55	0.74	0.78

**B. regional lymph node involvement (pN)**	**Accuracy**	**F1**	**Kappa**	**Recall**	**Precision**
ChatGPT	0.91	0.92	0.84	0.92	0.92
GPT-4-Turbo-1106-preview	0.95	0.95	0.91	0.95	0.96
NER Classifier	0.48	0.50	0.24	0.48	0.69
WordPiece tokenizer	0.70	0.71	0.46	0.70	0.74

**C. overall tumor stage**	**Accuracy**	**F1**	**Kappa**	**Recall**	**Precision**
GPT-3.5-Turbo-16K	0.76	0.76	0.61	0.76	0.77
GPT-4-Turbo-1106-preview	0.89	0.89	0.82	0.89	0.89
NER Classifier	0.30	0.28	0.06	0.30	0.51
WordPiece tokenizer	0.66	0.67	0.46	0.67	0.68

**D. histological diagnosis**	**Accuracy**	**F1**	**Kappa**	**Recall**	**Precision**
GPT-3.5-Turbo-16K	0.99	0.99	0.98	0.99	0.99
GPT-4-Turbo-1106-preview	0.99	0.99	0.98	0.99	0.99
NER Classifier	0.93	0.92	0.86	0.93	0.91
WordPiece tokenizer	0.93	0.92	0.86	0.93	0.92

**E. Average**	**Accuracy**	**F1**	**Kappa**	**Recall**	**Precision**
GPT-3.5-Turbo-16K	0.89	0.88	0.80	0.89	0.89
GPT-4-Turbo-1106-preview	0.94	0.94	0.89	0.94	0.94
NER Classifier	0.51	0.53	0.32	0.51	0.69
WordPiece tokenizer	0.76	0.76	0.58	0.76	0.78
